# Lessons learned from the frontline implementing person-centered practice: a practical pathway that addresses known challenges

**DOI:** 10.3389/frhs.2026.1820620

**Published:** 2026-07-13

**Authors:** Caroline Bills, Janette Gale

**Affiliations:** 1 HealthChange Associates, Melbourne, VIC, Australia; 2 Kangaroo Valley, Kangaroo Valley, NSW, Australia

**Keywords:** healthChange® methodology, implementation, person-centered care, person-centered care training, workforce development

## Abstract

Person-centred care (PCC) is widely endorsed as essential to high-quality healthcare yet consistent frontline delivery remains elusive. Existing frameworks largely describe what person-centred cultures should look like but provide limited operational guidance on how to enact person-centredness within real interactions under real conditions. This practice-informed perspective paper argues that broader frameworks need to be supported by practical methodologies that directly impact provider behaviour. Drawing on two decades of experience developing and implementing HealthChange® Methodology (HCM) with over 15,000 healthcare providers across three continents, we describe how a micro-engagement skills course transformed into a service delivery methodology for PCC and behaviour change. We openly acknowledge a conflict of interest as the commercial developers of HCM. HCM provides plain-language behavioural definitions, a consultation decision framework and audit tools that make person-centredness visible, measurable and improvable. Unlike frameworks operating at the organisational level, HCM defines a set of Person-Centred Practice Principles within a behaviour change framework to operationalise person-centredness within interactions across all health service roles. To our knowledge no other methodology integrates behaviour change science, health literacy guidance and a consultation decision framework into a single accessible operational system. We describe HCM's relationship to existing PCC frameworks, its distinctive features and how they address barriers consistently identified in the implementation literature. The evidence base is predominantly observational and practice-based. Independent research is needed and actively invited. PCC will become more widespread when broader frameworks are supported by practical, scalable methodologies that translate values into consistent everyday practice.

## 1 Introduction

“Practitioners want to do the right thing, not to make fools of themselves, and they need action plans to lean on, some role modelling and examples of HOW to work”. This observation from a recent report on the current state of Person-Centred Care evidence (1) captures a significant factor slowing PCC implementation. There is general agreement that consistent frontline delivery remains elusive (2, p.3) and there is a “need for much greater efforts to drive more practical, consistent and mainstream application” (3).

A challenge is to operationalise Person-Centred Care (PCC) with sufficient clarity to guide behaviour without sacrificing its necessity to be responsive. Existing frameworks largely describe what person-centred practice should look like but provide limited operational guidance on how to produce it under real conditions. Our contention is that PCC is likely to become more widespread if broader frameworks are supported by practical, existing methodologies impacting on behaviour.

This is a practice-informed, perspective paper written by the founder and current owner of HealthChange® Methodology (HCM) and we acknowledge a conflict of interest as a private operator and do not claim expertise outside of HCM. This paper describes the responses developed to address common barriers experienced by providers and managers when translating person-centred concepts into practice. We draw from decades of delivering training and consulting services to over 15,000 healthcare providers and collaborating with hundreds of health services, academics and researchers.

Our aim is to describe how a micro-engagement skills course transformed into a practice methodology for PCC and behaviour change. A detailed account of HCM theoretical architecture and contents is beyond the scope of this perspective paper, instead we focus on how it relates to existing PCC frameworks, its unique features and the barriers they address.

## 2 What facilitates implementation of PCC?

Lack of consensus on how to define, conceptualise, practise and measure PCC (4) contributes to confusion and inconsistent practice. Barriers exist at all levels of the system (5, 6), so effective responses need to be system-wide, involving workforce behaviour change and changes to service processes.

Key enablers are consistently identified. Workforce training is identified as both an implementation facilitator and a barrier: effective when experiential, reflective, and embedded in practice; ineffective when delivered as a one-off event disconnected from context (3, 5–7). Core competencies have been identified, although specific skillsets differ across roles (8, 9).

Frameworks such as the Person-Centred Curriculum Framework (PCCF) (2) and the Person-Centred Practice Framework (PCPF) (10), focus on the ethical and values foundations of person-centredness and guide application of learning into education and workplace settings. Key recommendations are clear definitions, a systems approach and shared language, however, do not address how providers integrate concepts with clinical tasks.

A positive workplace culture, strong leadership and knowledgeable change agents are consistently identified as critical enablers (6, 11, 12) and models such as Gothenburg Centre for Person-Centred Care (GPCC) model have been developed to assist implementation at the point of care (13). While significant advances have been made (14), person-centred practice is not yet widespread as usual practice (2, p.3, 15).

## 3 What is HCM?

HealthChange® Methodology is a service delivery methodology used to provide person-centred care so consumers feel heard, understood and are activated to understand and manage their health. It enables a consistency of approach across teams to embed person-centred work practices and behaviour change into interactions, workflows and service design. The purpose is to bridge the gap between theory and practice, giving providers and managers a shared operational system to improve care.

It provides plain-language behavioural definitions, a decision framework and audit tools to make person-centredness more easily visible, measurable, and improvable. Founded on meeting fundamental human needs and facilitating change, the practical content can operate at the level of the individual consultation, the team, and leadership. At the organisational level HCM can inform service workflow design, quality improvement activities and workforce development.

HCM is not a set of rigid protocols and the relational aspects can be used in isolation to develop relationships. It does not prescribe what should be said or coerce change and must be applied in line with the values of respect for persons, self-determination and mutual understanding that underpin person-centred practice (2, p.37).

Health professionals are tasked with facilitating consumer action, yet many are unsure how to do this while being person-centred. Unlike other frameworks operating at the organisational level, HCM defines a set of Person-Centred Practice Principles within a behaviour change framework to enact person-centredness within interactions at every level of the organisation. To our knowledge no other methodology integrates behaviour change science, health literacy guidance and a consultation decision framework into a single accessible operational system applicable across all health service roles.

The founder, Janette Gale, a Health Psychologist, initially collaborated with Deakin University in 2005 to create a course addressing an identified workforce gap: engagement and behaviour change skills. A company was founded in 2006 to continue providing training outside the University setting. However, teaching micro-skills in isolation resulted in low uptake into practice. Practitioners found abstract concepts difficult to transfer to clinical contexts, technical language unhelpful and isolated skills training gave no guidance on when or how to apply skills within existing workflows, correlating with challenges identified by others (6, 13).

## 4 Development

HCM was created between 2005 and 2012 by a multi-disciplinary team of clinicians (16). Being clinicians ourselves, we knew training had to be quickly understood and easily applicable. Plan, Do, Study, Act cycles involving thousands of providers across diverse roles and backgrounds tested and refined training content that has remained stable since 2012.

HCM's design is grounded in a formal literature review (17) that identified three evidence-based process sets: relationship-building processes drawing from self-determination theory and therapeutic alliance research; intention-forming processes from motivational and health belief models; and action-conversion processes from goal setting theory, implementation intentions and cognitive behavioural approaches. This synthesis identified that while many theories describe individual components of behaviour change, none provided a single integrated model that could be applied consistently across diverse clinical contexts. The critical insight was that three sets of sequential processes correspond to different stages of readiness: building the therapeutic relationship, forming an intention to act, and converting that intention into sustained action. While these processes are sequential, relationship-building is not confined to one stage, it remains the priority throughout every interaction.

The term “methodology” was chosen to indicate a bottom-up approach that emphasizes “how to” integrate the theoretical and values-based foundations of a person-centred approach into conversations, clinical interactions, and service design regardless of setting or social culture. While training is required to understand the contents, the clearly defined terms, the decision framework and the audit tools position it as a practical implementation and quality improvement framework for PCC.

While HCM was developed for application to clinical consultations, effective collaboration with any person or group requires the same processes and skills. When the context changes, the specific knowledge area and the desired target outcome alter, but not the core relational and behaviour change elements. This process consistency across contexts is what enables HCM to operate as a bottom-up methodology making it useful to all roles whenever interacting with a colleague, developing relationships or facilitating uptake of advice. Administration staff use it when sensing consumer hesitation to book appointments, clinical staff when outlining treatment options, managers to facilitate staff behaviour change.

We use the methodology to teach the methodology. Facilitators are required to demonstrate personal experience of practice change, wide-ranging implementation examples and competency with sceptical groups. This process maintains intervention fidelity and has been applied to training workplace champions in thirteen organisations to date.

## 5 The integrated suite of tools

*The Skills Layer* is a set of clearly defined Person-Centred Practice Principles and Essential Behaviour Change Techniques that operationalise interaction person-centredness. They are drawn from the literature but labelled in plain language to make them immediately recognisable and applicable. These brief examples illustrate how numerous concepts and techniques sit behind the simple label.

The First Ask, Then Offer Principle operationalises respect for autonomy, self-determination and personhood and prevents providers defaulting to “telling”. Nuanced skill is required to ask the right question at the right time, so this principle is supported by techniques that draw from appreciative inquiry techniques and motivational interviewing. This simple but challenging shift changes interaction dynamics from expert-led to collaborative. Providers can immediately relate to whether they use this principle or not and then focus on the behaviour to unlearn deeply habituated patterns.

Collaboration is not always easy. Call It As You See It, With Tact guides providers to have honest conversations about what they are observing and hearing, particularly around disengagement, low motivation or sensitive topics. Many providers avoid these conversations due to lack of skill or concern for the relationship. This principle encourages reflection and discussions about how to appropriately use the principle to raise difficult observations respectfully without judgement, which is essential for genuine person-centredness rather than avoidance.

The Four Aspects of Goal Setting is HCM's most structurally distinctive feature. It reframes goal setting to include four defined aspects: long-term clinical targets; broad treatment and lifestyle categories; short-term personalised goals; and motivational drivers linked to each. This structure ensures the person's perspective and readiness is prioritised at all times and addresses the common occurrence of goal setting, but little action.

Applying these principles in line with the values of respect for persons, self-determination and mutual understanding requires nuanced judgement and contextualisation. Practitioner reactions range from excitement to overwhelm (18). Consequently reflection, deliberate practice and facilitated discussion in a safe environment are essential components of HCM training.

### 5.1 The process layer

A conceptual Behaviour Change Pathway graphic is the foundational process tool guiding reorientation of care to meet people's information, motivational and action needs, consistent with self-determination theory (19) and the transtheoretical model of behaviour change (20). It visually depicts the order of processes enabling quick application of the concept of meeting people where they are at.

The Decision Line marks the point at which a person has decided to act. Above the line, the provider focuses on building importance and readiness; below it the focus shifts to building confidence and supporting action planning and self-regulation. Providers who move into goal setting before this threshold consistently find that plans are not followed. The time taken to progress through these processes is entirely dependent on the individual and their context. An underlying assumption is that not all people will cross the decision line.

**Figure 1:**
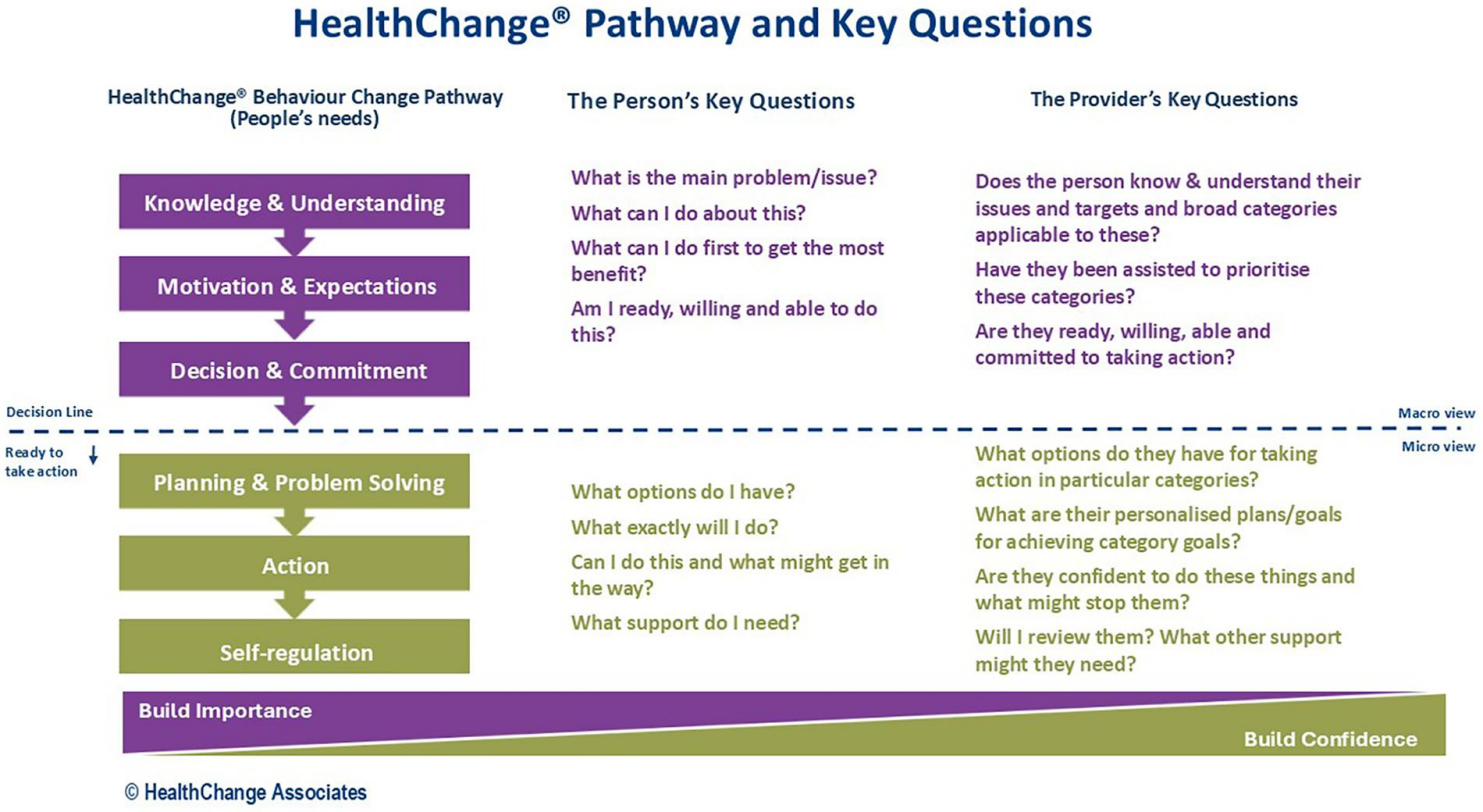
The HealthChange® Behaviour Change Pathway and Provider Key Questions.

Clinical effectiveness and time-efficiency is supported by a Consultation Decision Framework (CDF) guiding providers to align their conversation and clinical tasks with the person's needs at each process, actively identifying and addressing barriers as they arise (Figure 1). This combination of simple tools prompts providers to consider both what the person is asking themselves alongside what they should be asking.

A practical health literacy definition directly addresses the tension between evidence-based practice and person-centred care (21). It prompts providers to discuss relevant information people need at each stage rather than defaulting to generic information delivery. It can be applied within a consultation or more broadly as an activity for teams to translate clinical guidelines from the person's perspective.

*The Documentation and Audit Layer* support HCM application by closing the feedback loop and have been used in qualitative and implementation studies (22, 23). They are not comprehensive measures of PCC but provide individuals and teams with a simple method to reflect on confidence and competence applying HCM skills and processes.

## 6 How HCM complements existing frameworks and addresses known implementation challenges

HCM complements rather than competes with existing frameworks, models and training programs. The operational layers distinguish HCM as an implementation methodology. Training, introducing all or a subset of elements, is the primary delivery mechanism through which providers and managers develop working knowledge of HCM, enabling organisations to demonstrate how they meet national quality standards (24, 25). The following observations are offered from our perspective as a basis for further dialogue.

Successful training needs to address three questions: What is involved? How do I do it? How do I know I am doing it? HCM addresses each of these issues and our training approach aligns with the PCCF's four learning principles of transformative, co-constructed, relational and pragmatic (26).

HCM is not designed to provide deep training in every skill it encompasses. Rather it provides an operational framework that gives the skills and values of person-centredness, communication, shared decision-making, behaviour change and health literacy their home and sequence within interactions. It can therefore be used alongside other courses. Providers who have completed other training report that HCM helps them apply their skills more effectively.

HCM is delivered primarily in workplace settings with emerging uptake in tertiary education. HCM training is not delivered as a one-size-fits-all program. Training and implementation activities are co-constructed with organisations to suit their workforce, readiness and budget, with options ranging from single modules to full implementation support to embed HCM into workflows, including Peer Champion and Train-the-Trainer programs.

## 7 Defining person-centred practice: HCM's contribution to conceptual clarity

The Person-Centred Approaches capability framework (9) identifies a core skillset and three steps: conversations to engage, to enable and support and to collaboratively prioritise complexity and risk. HCM includes this broad skillset and provides more specific operational guidance (Figure 1).

HCM's defined elements directly address the conceptual confusion and training variability identified as a significant barrier to applying concepts into practice (1) The adaptable framework enables a consistency of approach across health service teams to embed person-centred work practices into clinical consultations, care planning, discharge planning, disease management, health promotion, rehabilitation, return to work and other health services. Attending HCM training alone was identified as a key enabler of implementation of an osteoarthritis management program (27). The CareFirst program continues to embed HCM after a pilot study demonstrating clinical improvements and strong acceptance by both patients and providers (28).

HCM's defined skills and processes enable providers to identify more specifically what they are doing well and where there are gaps. One study demonstrated that training in these principles changes provider attitudes and positively impacts practice (18), and a participant quote captures the shift in awareness this produces: “*I probably thought I was doing PCC but realized that perhaps I wasn't.”.*

## 8 Operationalising person-centred practice: tools for the point of care

HCM fits primarily within the PCPF's prerequisites domain, relating to professional competence and interpersonal skills and the person-centred processes domain, providing the operational tools that make those domains actionable at the point of care. The definitions and audit tools provide a quick feedback mechanism and concrete reference points to support the ongoing theoretical and values discussions required for organisational maturity. The health literacy definition directly addresses the common practitioner question: “How do I integrate these skills with my clinical tasks and duty of care?” HCM has been used to operationalise the translation of clinical guidelines into person-centred practice, osteoarthritis interventions (29, 30).

When embedded, HCM provides a mechanism to achieve person-centred process consistency while allowing content and context adaptability. A new staff member oriented to a multidisciplinary primary care clinic observed three client interactions with different team members. Afterwards she asked her manager: “What system are you using? I have just seen three different consultations with different people but there is a consistent conversational style and underlying process going on.” What she observed was HCM in action: variability in content, consistency in person-centred application. This consistency is not produced by rigid protocols but through the framework that enables providers to respond in the moment.

In 2012, Alberta Health Services selected HCM to embed PCC into Primary Care services province-wide. HCM was selected for its respect for existing provider skills, its practical point-of-care approach and its collaborative implementation model focused on building workforce capacity and reorienting service processes.

## 9 Measuring and sustaining person-centred practice: closing the feedback loop

Both the GPCC model and HCM apply at the point of care, guide professionals without imposing rigid protocols and address documentation. HCM provides clinical integration and feedback tools that can quickly alert managers to gaps in care. Its behaviour change elements are relevant across all roles and begin to address an identified knowledge gap in change management skills (1, p.44). When leaders attend the same training as frontline staff, they gain the shared language needed to coach, supervise and reinforce person-centred practice expectations and send a clear message that PCP is an organisational expectation. Manager training ensures they inform discussion and minimises the risk of a compliance approach (1, p.84).

Faster adoption of PCC is likely when providers and managers are empowered to use the same tools across teams with simple methods to track impact and increase accountability (1, p.88, 6). Skills learned through HCM travel with providers across roles and organisations meaning training investment compounds rather than being lost through staff turnover. A consistent implementation methodology also addresses the issues of variation in training and implementation strategies (13).

## 10 Limitations and conclusion

This paper is a practice-informed perspective and we acknowledge a conflict of interest as commercial providers of HCM. The evidence base is predominantly observational and practice-based with no independent randomised controlled trial of HCM conducted to date. HCM provides process guidance but does not prescribe cultural content or clinical specifics which remain the provider's responsibility. Risks include superficial adoption without genuine values engagement and implementation without adequate manager involvement. Adoption has been constrained by limited priority given to implementation methodology at the commissioning level. We invite independent research to address these gaps.

HCM offers a practical strategy that balances simplicity and comprehensiveness, complementing the progress made so far. Practitioners want to do the right thing, not to make fools of themselves, but need practical guidance. The field will advance faster if institutions and practice-based organisations work together so we extend an open invitation to collaborate.

## Data Availability

The data analyzed in this study is subject to the following licenses/restrictions: Course feedback data is held by HCA. Requests to access these datasets should be directed to Caroline Bills, c.bills@healthchange.com.
